# Influence of Hybrid Surface Modification on Biocompatibility and Physicochemical Properties of Ti-6Al-4V ELI Titanium

**DOI:** 10.3390/jfb15030052

**Published:** 2024-02-20

**Authors:** Anna Woźniak, Weronika Smok, Janusz Szewczenko, Marcin Staszuk, Grzegorz Chladek

**Affiliations:** 1 Materials Research Laboratory, Faculty of Mechanical Engineering, Silesian University of Technology, Konarskiego 18A Street, 44-100 Gliwice, Poland; 2 Department of Engineering Materials and Biomaterials, Silesian University of Technology, Konarskiego 18A Street, 44-100 Gliwice, Poland; weronika.smok@polsl.pl (W.S.); marcin.staszuk@polsl.pl (M.S.); 3 Department of Biomaterials and Medical Devices Engineering, Faculty of Biomedical Engineering, Silesian University of Technology, Franklina Roosevelta 40 Street, 41-800 Zabrze, Poland; janusz.szewczenko@polsl.pl

**Keywords:** Ti-6Al-4V, corrosion, EIS, SEM, electrospinning, nanofibers, PCL

## Abstract

Titanium-based materials are the most widely used materials in biomedical applications. However, according to literature findings, the degradation products of titanium have been associated with potential allergic reactions, inflammation, and bone resorption. The corrosion process of Ti-6Al-4V in the human body environment may be exacerbated by factors such as reduced pH levels and elevated concentrations of chloride compounds. Coatings made of biopolymers are gaining attention as they offer numerous advantages for enhancing implant functionality, including improved biocompatibility, bioactivity, wettability, drug release, and antibacterial activity. This study analyzes the physicochemical and electrochemical behavior of the Ti-6Al-4V ELI alloy subjected to PCL and PCL/TiO_2_ deposition by the electrospinning method. To characterize the polymer-based layer, tests of chemical and phase composition, as well as surface morphology investigations, were performed. Wetting angle tests were conducted as part of assessing the physicochemical properties. The samples were subjected to corrosion behavior analysis, which included open circuit potential measurements, potentiodynamic tests, and the electrochemical impedance spectroscopy method. Additionally, the quantification of released ions post the potentiodynamic test was carried out using the inductively coupled plasma atomic emission spectrometry (ICP–AES) method. Cytotoxicity tests were also performed. It was found that surface modification by depositing a polymer-based layer on the titanium substrate material using the electrospinning method provides improved corrosion behavior, and the samples exhibit non-toxic properties.

## 1. Introduction

In medical applications, stainless steel, cobalt-based alloys, and titanium and its alloys are often used for long-term implants. Each is characterized by distinct properties and applications. Stainless steel, especially type 316 L, is used in orthopedic applications such as joint replacements, bone plates, and screws. However, corrosion-resistant stainless steel may exhibit wear and tear over time. Additionally, stainless steel is characterized by the lowest corrosion resistance in the metal biomaterials group. Its magnetic properties can interfere with medical imaging [1,2]. In turn, cobalt-based alloys, known for their high strength, corrosion resistance, and biocompatibility, find application in orthopedic implants, dental prosthetics, and cardiovascular devices. Despite their advantages, the density of cobalt-based alloys can be high, resulting in increased weight in implants. Moreover, some individuals may experience allergic reactions to cobalt [1,3]. While stainless steel and cobalt-based alloys have their applications, titanium alloys, despite potential cost considerations and a challenging fabrication process, stand out due to their superior biocompatibility, mechanical properties, corrosion resistance, and ability to promote osseointegration [4,5]. Ti6-Al-4V is characterized by the best biocompatibility in the metal biomaterials group, thereby reducing the likelihood of negative reactions within the human system. Its surface properties facilitate the formation of a biologically inert oxide layer, promoting favorable interactions with the surrounding tissues [6]. In addition, titanium-based materials exhibit good corrosion resistance due to their ability to spontaneously form a thin and stable protective oxide layer, which constitutes a compact and dense kinetic barrier for extensive corrosion [7]. Additionally, titanium alloys exhibit an excellent strength-to-weight ratio and their low density results in lightweight implants, and they actively promote osseointegration, ensuring long-term stability. These attributes collectively position titanium, especially Ti-6Al-4V, as the preferred material for medical implants where long-term stability and success are paramount. Despite its numerous advantages, Ti-6Al-4V does pose certain challenges in biomedical applications. Iwabuchi et al. [8] and Beake et al. [9] pointed to the poor wear resistance of Ti-6Al-4V, which was lower than that of most metallic biomaterials. Wear debris generated during the abrasion process can lead to the release of particles into the surrounding biological environment. While the biological response to these particles varies, the potential for adverse reactions and inflammation exists [10]. In addition, abrasion or frictional forces may compromise the integrity of the protective passive layer, exposing the biomaterial to corrosion. Moreover, in aggressive biological environments, such as those with high chloride concentrations, the passive film may become susceptible to breakdown. Corrosive attack can compromise the structural integrity of implants, potentially leading to mechanical failure [11].

Presently, surface laser texturing is acknowledged as a highly effective method for improving material performance [12,13]. Laser texturing proves to be a precise, accurate, reproducible, and environmentally friendly technique for modifying surfaces. Literature findings reveal that the laser-texturing process not only results in an enhancement of tribological behavior attributed to improved lubrication [14,15,16,17,18,19,20,21], but also leads to superior microbiological properties owing to the contact guide effect [22,23].

It is worth paying attention to coatings made of biopolymers, because they offer many benefits for increasing the functionality of the implant, including improved biocompatibility, bioactivity, wettability, drug release, and antibacterial activity [24,25]. The most commonly used methods for applying polymer coatings on biomedical alloys include dip-coating [26,27], spin-coating [28], electrochemical assembly [29], and electrospinning from solution [30]. Electrospinning is a simple, low-cost, and repeatable method of manufacturing homogeneous, porous nanofiber networks that mimic the extracellular matrix (ECM) of the cell with their morphology, which additionally improves their bioactivity [31,32]. Previous research indicates that this technique is successfully used to coat biomedical alloys with fibrous layers [33,34,35]. Karthega et al. [36] showed that polycaprolactone/titanium dioxide (PCL/TiO_2_) nanofibers deposited by electrospinning on the surface of the AM50 Mg alloy not only improve the corrosion resistance of the alloy, but are also beneficial for cell proliferation. Kim et al. [37] significantly improved the biocompatibility of MC3T3-E1 osteoblasts (an osteoblast precursor cell line derived from mouse calvaria) on the AZ31 Mg alloy by covering its surface with polycaprolactone/zinc oxide (PCL/ZnO) nanofibers via electrospinning. The electrospinning method also allows for the deposition of nanofibers on the Ti-6Al-4V alloy, as reported by Rajabi et al. [38] and Camargo et al. [39]. E.R. Camargo et al., on a previously prepared surface of a commercial Ti-6Al-4V alloy, built a network of poly(methyl methacrylate) (PMMA) nanofibers and functionalized PMMA-OH nanofibers by electrospinning. It was observed that the use of a polymer coating significantly improved the adhesion and proliferation of fibroblasts. Rajabi et al. performed a two-stage modification of the surface of the Ti-6Al-4V alloy: first, the surface was modified with the Nd:YAG (neodymium-doped yttrium aluminum garnet) laser; then, drug-loaded poly(vinyl alcohol) (PVA) nanofibers with vancomycin were deposited on it. The applied surface modifications improved biocompatibility, cell viability, and adhesion, as well as extended the drug release rate.

This study aims to investigate the influence of a hybrid surface modification through laser texturing and the deposition of a polycaprolactone-based (PCL) nanofiber layer using the electrospinning method on the physicochemical properties of the Ti-6Al-4V titanium alloy. The use of polycaprolactone (PCL) nanofiber layers shows significant promise for drug delivery post-implantation in the human body. Electrospinning enables the creation of nanofibrous structures with a high surface area, providing an optimal platform for controlled drug release. The potential impact of this approach on post-implantation therapeutics is substantial, offering controlled and sustained drug release, minimizing side effects, and improving overall treatment outcomes. Combining nanofiber layers deposited via the electrospinning method with a laser-texturing process may ensure the increased biocompatibility of biomaterials and overcome the primary usage limitations of the Ti-6Al-4V alloy. Nevertheless, the initial impact of this proposed hybrid surface modification on the physicochemical properties, especially the wettability and corrosion resistance, of the titanium alloy must be rigorously evaluated. This study represents the initial step in the development of new biomaterials for long-term implants, ensuring the potential for controlled drug release. The integration of PCL nanofibers and laser texturing offers a synergistic approach to addressing biocompatibility concerns and usage limitations, laying the foundation for advancements in the field of implantable medical devices. 

In this work, surface morphology analysis was conducted using scanning electron microscopy and confocal microscopy. Particular attention was devoted to the assessment of the wettability and corrosion behavior of the modified biomaterials. Contact angle measurements and surface free energy calculation were performed to investigate the chemical character of the samples. The corrosion behavior was analyzed by a potentiodynamic test, supplemented by an electrochemical impedance spectroscopy test. In addition, biological (toxicity) properties were evaluated. 

## 2. Materials and Methods

The Ti-6Al-4V samples in cubic shape (φ 14) were wet-ground (using silicon carbide SiC paper with grit of P500 to P4000), and then polished using a colloidal silica suspension (OP-U) with a particle size of 0.04 μm. The mean roughness of the samples was Ra = 0.52 µm.

Next, samples were subjected to surface modification. The surface texturing process was accomplished using an A-355 picosecond laser system (Oxford Lasers Ltd., Didcot, UK), utilizing a 355 nm wavelength diode-pumped solid-state picosecond laser which generates 5–10 ps pulse durations of 120 µJ average power at 400 Hz pulse frequency. The system of pulsed laser beams guarantees high energy densities and the ability to perform ablation (atoms evaporate layer by layer due to the strength of bonds being decreased between the particles). Micromachining system guarantees average power of 24 mW. The laser beam intensity distribution is Gaussian. The experiment was performed in air at atmospheric pressure. The path of laser texturing was a system of grooves, which formed a truss shape. The Cimita software (2013 version, Oxford laser, Didcot, UK), integrated into the micromachining system, was used to design the laser pattern and process parameters setup. The process parameters are as follows: number of the passes of the laser beam (N)—2, laser scan speed—1 mm/s, beam width—30 µm, and beam quality factor (M^2^) < 1.2.

**Figure 1:**
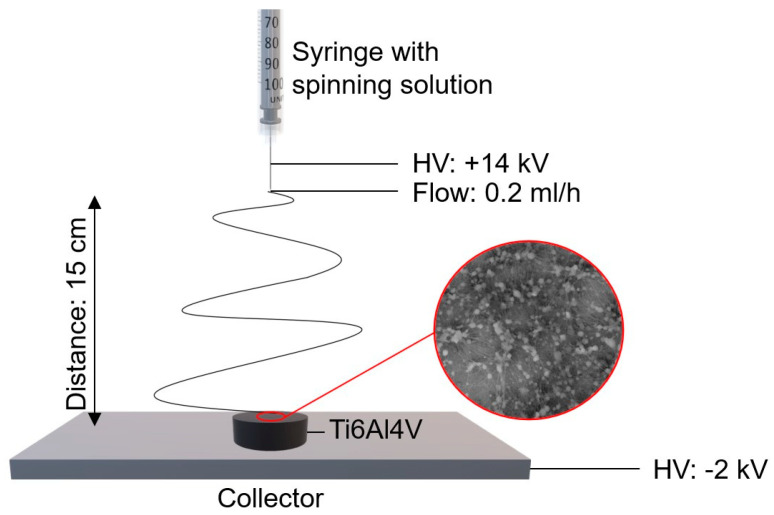
Scheme of electrospinning process.

To prepare polymer and composite coatings on the Ti-6Al-4V alloy, the electrospinning form solution method was used. The first stage was the preparation of spinning solutions, for which the following reagents were used: polycaprolactone (PCL purity average Mw 45000, Sigma Aldrich, St. Louis, MO, USA), formic acid (purity 96%, Sigma Aldrich), acetic acid (purity 99%, Sigma Aldrich), and TiO_2_ nanoparticles (purity 99.5%, Bionovo). To prepare PCL solution, acetic acid was mixed with formic acid in a 3:1 ratio; then, 3 g of PCL in the form of granules was added and left on a magnetic stirrer to mix for 24 h. The spinning solution of PCL/TiO_2_ nanofibers was prepared by adding 0.06 g of TiO_2_ to the above-mentioned acid mixture and sonicated for 15 min to break down the nanoparticle agglomerates. Then, 3 g of PCL were added and left on a magnetic stirrer for 24 h until a homogeneous solution was obtained. The next stage was to subject the solutions to the electrospinning process using the FLOW device—Nanotechnology Solutions Electrospinner 2.2.0–500 (Manufacturer Yflow Nanotechnology Solutions, Malaga, Spain) with these constant parameters for both samples: distance, voltage, and flow rate of 15 cm, 16 kV, and 0.2 mL/h, respectively. The deposition time of the fibrous layer for each sample was 15 min (Figure 1). These parameters were selected experimentally to ensure the stability of the electrospinning process.

The microstructure and morphology of the coatings obtained were examined using high-resolution scanning electron microscopy (SEM), the FEI Supra 35 (Zeiss) type, equipped with energy dispersive X-ray spectroscopy (EDX).

The wettability of tested samples was evaluated by contact angle (θ) measurements (sitting drop method), and surface free energy (γ) calculations using Owens–Wendt methods were performed. The test stand was equipped with a Surftens Universal goniometer (OEG, Frankfurt, Germany) and PC with Surftens 4.5. Distilled water θ_w_ (POCH S.A., Gliwice, Poland) and diiodonomethane θ_d_ (Merck, Warsaw, Poland) served as the measured liquids (drop volume of 1.0 µm^3^). The measurements were conducted at room temperature T = 23 ± 1 °C over time t = 60.

Pitting corrosion resistance tests were performed using the potentiodynamic method. The test stand comprised of an Atlas 0531 EU potentiostat (ATALS-SOLLICH, Rębiechowo, Poland) and three-electrode cell. A platinum rod (auxiliary electrode) and a silver chloride electrode Ag/AgCl electrode (reference electrode) were used for the tests. Corrosion testing commenced following 3600 s of open-circuit potential stabilization (E_ocp_). The scan rate was configured at 0.175 mV/s.

Electrochemical impedance spectroscopy (EIS) tests were performed using an identical test stand to that of the corrosion resistance test. The test was conducted in the frequency range of 10^4^ to 10^−3^ Hz. The amplitude of the sinusoidal voltage of the excitation signal was 10 mV. In the study, the impedance spectra in the form of the Nyquist and Bode diagrams were determined, and, next, obtained data were adjusted to the equivalent circuit using the least-squares method. All electrochemical analyses were carried out in a Ringer solution of the following chemical composition: NaCl—8.6 g/dm^3^, KCl—0.3 g/dm^3^, and CaCl_2_·2H_2_O—0.33 g/dm^3^, at T = 37 ± 1 °C.

The content of the titanium, aluminum, and vanadium in Ringer solutions after the immersion test was determined using inductively coupled plasma atomic emission spectrometry (ICP-AES). A Varian 710-ES spectrometer, equipped with a OneNeb nebulizer and twister glass spray chamber, was used. The parameter is given in Table 1. 

The cytotoxicity of the modified samples was examined with a 3-[4,5-dimethylthiazol-2-yl]-2,5 diphenyltetrazolium bromide (MTT) test. The HCT116 cancer cells (obtained from the American Type Culture Collection) were used for the test. The cell lines were treated with 10% fetal bovine serum (Eurx) supplemented with 1% antibiotic antifungal solution. The cells were seeded onto the tested samples and incubated for 72 h at 37 °C in a humidified atmosphere of 5% CO_2_. Next, the culture medium was removed, and, after trypsin neutralization, the cell suspension was centrifuged (2000 rpm, t = 3 min, T = 23 ± 1 °C), and the cell pellet in MTT solution was resuspended. After incubation (t = 3 h), the MTT solution was removed. In effect, obtained formazan was dissolved in C_3_H_7_OH:HCl. After that, the optical density at 550 nm with a reference wavelength of 670 nm was measured using an ELISA reader. The mean absorbance in control wells was considered to be 100% viable cells.

The names of the samples subjected to the test are given in Table 2.

## 3. Results

### 3.1. Surface Morphology Analysis

**Figure 2:**
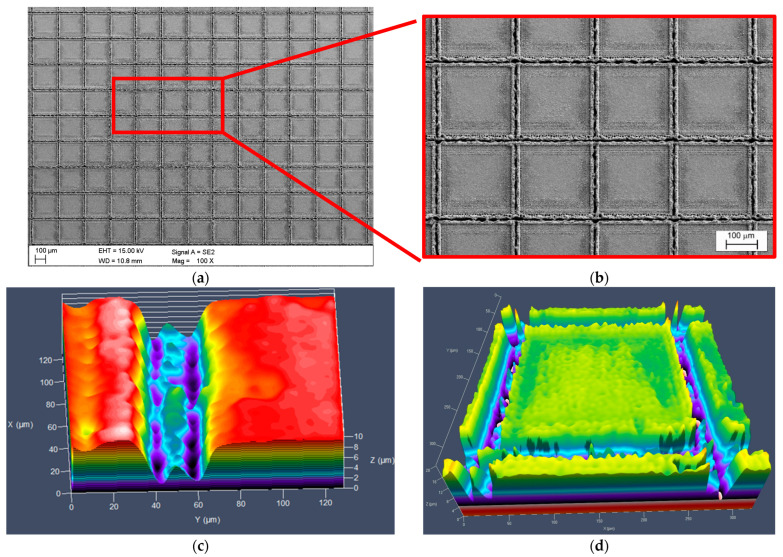
Results of microscopic observation for samples after laser-texturing process (S_tex): (**a**,**b**) SEM; and (**c**,**d**) confocal microscope.

The surface morphology of the samples after the laser-texturing process was characterized using a scanning electron microscope and a confocal microscope, as depicted in Figure 2. For the S_tex sample groups, cross-like micro-groove patterns were observed, which are characteristic features of photothermal ablation. The average width between micro-grooves was 242 ± 4 µm, consistent with the designed laser texture pattern. Additionally, a small amount of deposition, called micro-bugle or micro-crown, was noted on the edge of the micro-grooves of the titanium alloy. The height of the micro-bugle measured from confocal microscopy observation was 5.2 ± 0.5 µm (Figure 2c). This phenomenon could be attributed to the formation of a remelting layer on the titanium alloy surface during the thermal effects of laser processing. Wang et al. [7] and Huerta-Murillo et al. [40] also reported the existence of micro-bugles along the laser texture pattern. The groove depth was 9.4 ± 0.4 µm (Figure 2b). Additionally, the roughness of the central area of the laser pattern (single square) was Sn = 270 nm.

**Figure 3:**
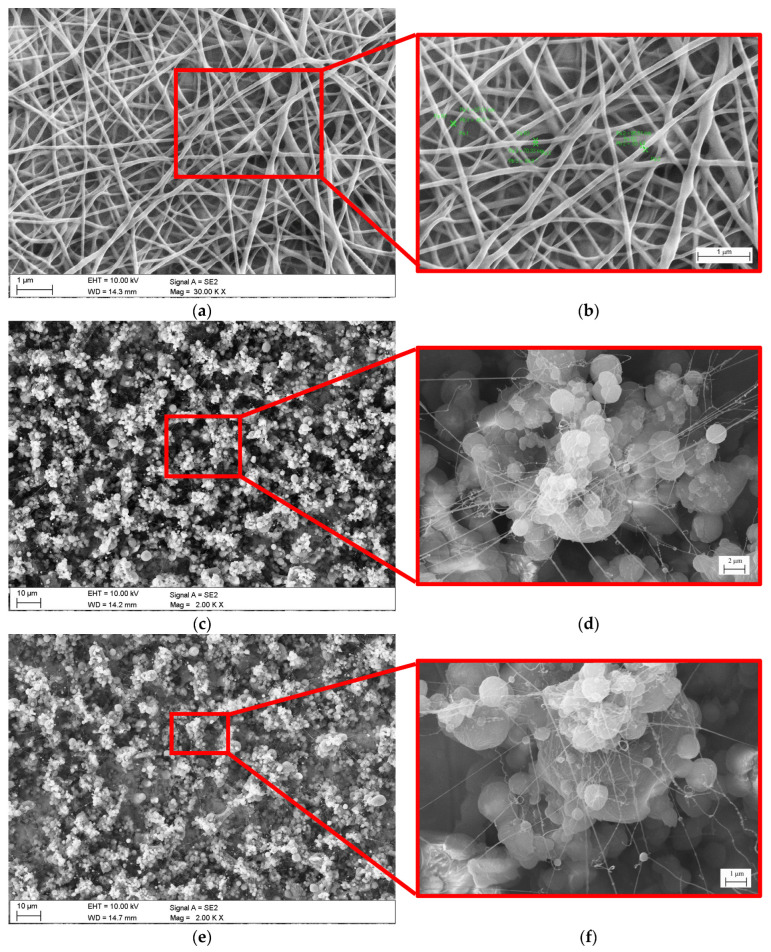

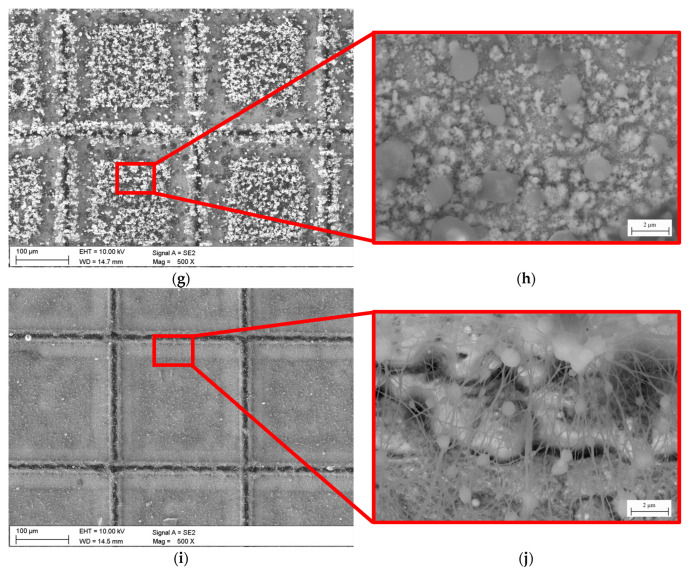
Results of samples surface morphology (SEM): (**a**,**b**) PCL nanofibers on aluminum foil, (**c**,**d**) samples with PCL nanofibers (S_PCL), (**e**,**f**) samples with PCL nanofibers with the addition of TiO_2_ particles (S_PCL/TiO_2_), (**g**,**h**) samples after laser-texturing process with PCL nanofibers (S_tex/PCL), and (**i**,**j**) samples after laser-texturing process with PCL nanofibers with the addition of TiO_2_ particles (S_tex_PCL/TiO_2_).

The analysis of the morphology of electrospun PCL and PCL/TiO_2_ nanofibers was performed based on SEM images (Figure 3). At first, to select the electrospinning process parameters, the PCL nanofibers were deposited on the aluminum foil. The PCL nanofibers deposited on the aluminum foil were homogeneous, had a smooth surface, and did not show any defects, e.g., beads and spindles, and their average diameter was 85 nm (Figure 3a,b). The absence of defects indicates that the parameters of the spinning solution were appropriate. The PCL nanofibers deposited on a Ti-6Al-4V substrate both before and after texturization were characterized by the presence of beads whose diameter ranged from 50 to several micrometers, while the diameter of the fibers themselves in both samples was approximately 60 nm (Figure 3c,d,g,h). Moreover, PCL/TiO_2_ nanofibers deposited on Ti-6Al-4V substrates had visible beads with a diameter reaching several microns, while the average fiber diameter was 33 and 73 nm for the unmodified and textured substrates, respectively. Comparing Figure 3g and Figure 3i, it was also found that the addition of TiO_2_ to PCL had a positive effect on the uniform coverage of the textured substrate with nanofibers.

The presence of beads and spindles in nanofibers electrospun onto a Ti-6Al-4V substrate was also observed by Camargo et al. [39] and Santhosh et al. [41], who studied the PMMA and hydroxyapatite–polysulfone coating, respectively. This type of defect appears mainly as a result of a too-low polymer concentration in the spinning solution or a too-low molecular weight of the polymer; however, in this study, nanofibers deposited from the same spinning solution deposited on the aluminum foil showed no defects, which suggests that the morphology of the fibers was largely influenced by the substrate. As indicated by [42,43,44], electrospinning on a non-conductive substrate such as Ti-6Al-4V is difficult due to electric field distortion, which destabilizes the polymer jet, adversely affecting the morphology of the obtained fibers.

### 3.2. Wettability Test

**Figure 4:**
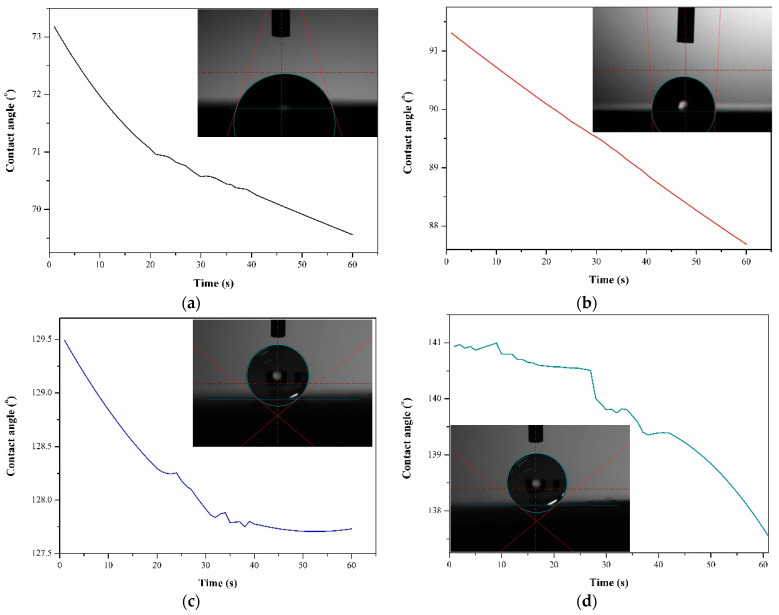

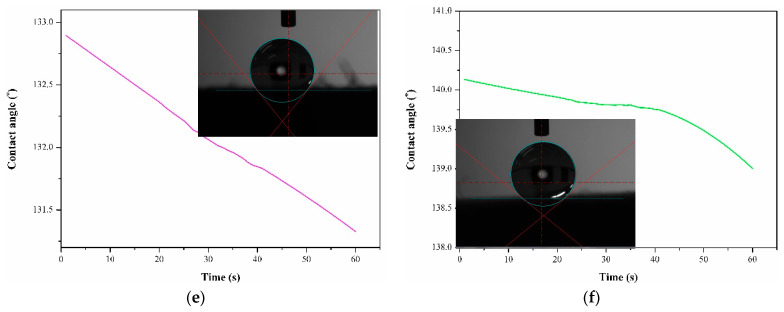
Results of wettability test: diagram of contact angle changes in time function and examples image of water drop on sample’s surface: (**a**) S_is, (**b**) S_PCL, (**c**) S_tex, (**d**) S_PCL/TiO_2_, (**e**) S_tex/PCL, and (**f**) S_tex/PCL/TiO_2_.

The results of the wettability test are presented in Table 3. The diagram illustrating changes in the contact angle of distilled water over time and example images of drops placed on the tested sample surfaces are shown in Figure 4.

Significant differences in the values of wettability were observed between the untreated samples and samples after surface modification. It was found that laser texturing and layer deposition by electrospinning have a significant effect on the contact angle of T Ti-6Al-4V-based biomaterial. For the untreated (S_is) samples group, the average water contact angle for the samples was approximately 68°, signifying a hydrophilic nature of the surface (Θ < 90°). The observed wetting state is characteristic of titanium surfaces stored under atmospheric conditions (air) [45]. Moreover, Melo-Fonseca et al. [46] and Reggio et al. [47] reported the hydrophilic nature of mechanically polished Ti-6Al-4V alloy. In contrast, for the samples after surface modification, a switch from hydrophilic to hydrophobic behavior is evident. For the S_tex samples group, the average value of the water contact angle was 95°, reaching the threshold for the transition from the hydrophilic to the hydrophobic state (where the hydrophilic state is <90° < hydrophobic state). The highest values were obtained for the samples with PCL and composite PCL-TiO_2_ layers, irrespective of the surface condition of the substrate material (polished or textured), with mean values falling within the range of 128–137°. According to the literature, electrospinning is a well-known method that enables the production of hydrophobic, ultrathin fibers with micrometer and sub-micrometer diameters from various polymeric materials. The electrospun fibers, due to their size, guarantee a single-length scale of roughness for superhydrophobicity. Sheela et al. [48] also indicated the hydrophobic nature of polycaprolactone electrospun membranes, reporting a mean value of 129°, which is similar to the values presented in our work. The addition of TiO_2_ particles to the PCL fibers led to a slight increase in the water contact angle. Generally, TiO_2_ particles exhibit hydrophilic behavior due to their polar nature. However, when introduced into a nanofiber matrix, they can influence the overall wetting properties. The inorganic TiO_2_ was easily covered by airborne organic contaminations and, in effect, demonstrate hydrophobic surface properties. Moreover, incorporating TiO_2_ particles into a nanofiber matrix can alter the composite material’s wettability, and this transition may depend on factors such as particle concentration and dispersion. Sahoo et al. [49] demonstrated that, with an increase in the titanium content from 1 to 2wt.% in the polyvinylidene fluoride (PVDF) matrix, the contact angle increased from 124 to 129°. Additionally, they indicated that a titanium dioxide concentration of more than 2wt.% strongly affects increased wettability. This finding confirms our investigation, where the TiO_2_ concentration in PCL fiber material was 2%.

The values of surface free energy of the S_is and S_tex samples were similar and fell within the range of 37–39 mJ/m^2^. The calculated values for samples with a nanofiber layer were approximately three times higher compared to the S_is and S_tex sample groups. In addition, for the samples with a hybrid surface modification, the S_tex/PCL/TiO_2_ SFE calculation was impossible, due to the extremely low value of the diiodomethane contact angle—close to 0°. Additionally, all tested samples demonstrated a greater affinity for apolar groups than polar ones, as evidenced by higher values of the apolar components of surface free energy (SFE). However, the apolar components of samples with PCL and PCL/TiO_2_ layers were four and two times higher compared to those for samples in the initial state and after the laser-texturing process, respectively. Dispersive interactions are primarily driven by van der Waals forces and occur between nonpolar molecules or regions of molecules. Dispersion forces are generally weaker than polar forces but can be more prevalent across a surface.

The wettability of materials is regulated by their chemical composition and surface morphology, including surface roughness. This property is directly manifested in contact, as described by the Young formula:

(1) $$\cos\Theta =r\frac{\gamma_{sg}-\gamma_{sl}}{\gamma_{lg}}=r\cdot cos\Theta ,$$

where:

γ—surface tension, s—solid state, g—gas, and l—liquid;

Θ—water contact angle (°);

r—roughness ratio (r = 1—smooth surface, and r > 1—rough surface).

From (1), it is observed that the contact angle increases with an increasing roughness ratio value if the contact angle on a smooth surface is more than 90°. The laser-texturing process leads to Ti-6Al-4V titanium alloy surface development at the micro- and nanoscale. Moreover, layers consisting of nanofibers of PCL and PCL/TiO_2_ deposited by electrospinning drastically affect the overall surface. 

**Figure 5:**
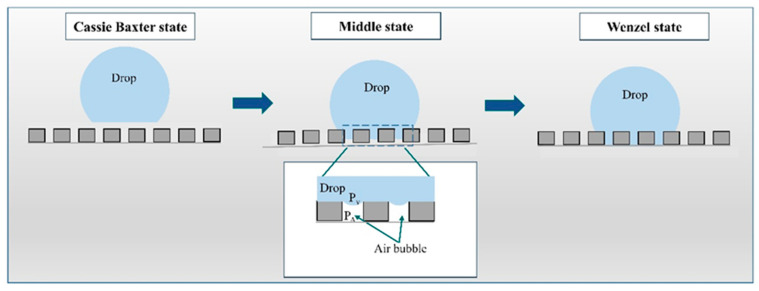
Scheme of wettability states.

According to the literature data, in general, three models that describe the wetting behavior on the rough or restored surface can be distinguished (Figure 5): (I) Wenzel, (II) Cassie–Baxter, and (III) the middle state, which is a transitional state between the Wenzel and Cassie–Baxter states. In the Wenzel state, a liquid completely wets a rough surface, filling the surface asperities. The liquid wets the roughness at a microscopic level, increasing the apparent contact area. In the Cassie–Baxter state, the liquid only partially wets the rough surface, with air trapped within the surface asperities. The air exhibits absolutely hydrophobic properties with a contact angle of 180° [50]. The apparent contact area is reduced, leading to a higher contact angle compared to the Wenzel state. In the middle state of wettability, the liquid partially occupies the surface features, creating a mixed wetting behavior. For the samples after surface modification, the wettability state probably occurs at the Cassie–Baxter state or the middle state of wettability. Moreover, external influences, such as the absorption of hydrocarbons from the atmosphere, may have impacted the results, as reported by Yamauch et al. [51] and Khan et al. [52].

### 3.3. EIS Test

**Figure 6:**
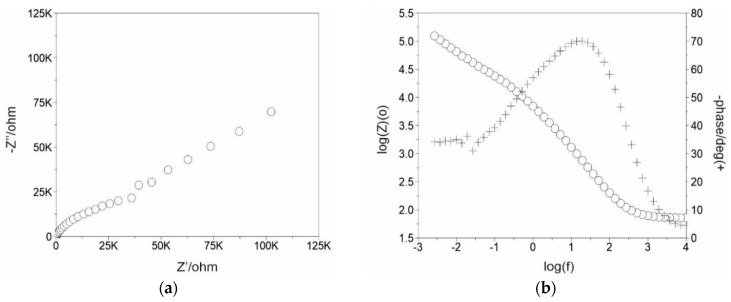

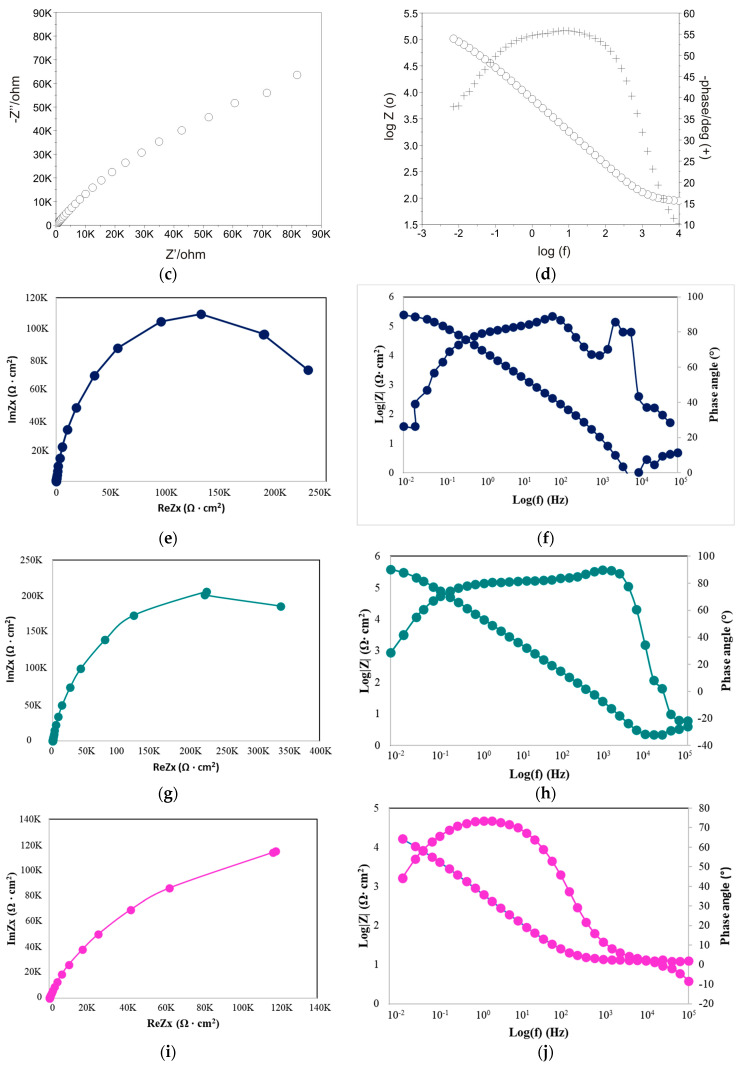

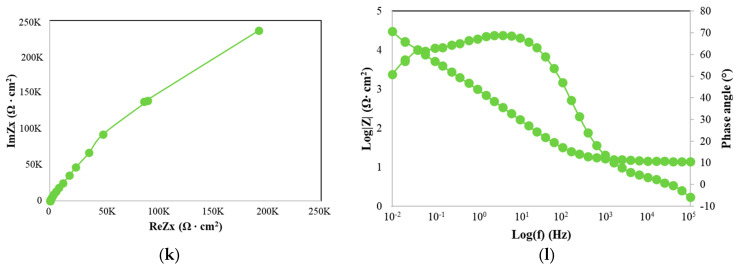
Results of EIS test in form of Nyquist and Bode diagram for the following: (**a**,**b**) S_is, (**c**,**d**) S_tex, (**e**,**f**) S_PCL, (**g**,**h**) S_PCL/TiO_2_, (**i**,**j**) S_tex/PCL, and (**k**,**l**) S_tex/PCL/TiO_2_.

Figure 6 shows the EIS spectra recorded for both sample groups in the form of Nyquist and Bode plots.

Based on the obtained results, it can be seen that the impedance response of the titanium alloy has remarkably changed after surface modification by laser texturing and PCL-based nanofiber layer deposition in the corrosive solution. The Nyquist plots (Figure 6a,c,e,g,i,k) presented fragments of semi-circles, which is a typical response of a thin layer. In addition, from Nyquist-impedance plots, it can be seen that only for the samples after the laser-texturing process was the radius of curvature lower than that of the untextured surface. For other samples, diagrams show a larger radius of the semicircle than that of the samples in the initial state. The diameter of the semicircle increases in the order of S_PCL < S_tex/PCL < S_PCL/TiO_2_ < S_tex/PCL/TiO_2_. According to the literature data [7,53,54], and our previous reports [6,55,56,57], the larger the radius of curvature in the Nyquist plots, the better the corrosion resistance of the sample surface. This suggests that surface modification by electrospinning provides superior corrosion resistance compared to the textured surface. Additionally, the results depicted in the Bode diagrams corroborate those of the Nyquist plots. The lowest value of the maximum phase displacement, over a broad range of frequencies, was observed for the S_is and S_tex sample group, with mean values of 65 and 50°, respectively. In contrast, for other samples, the phase displacement ranged from 70 to 95°, indicating that samples with PCL and PCL/TiO_2_ layers exhibit a higher impedance and better corrosion resistance.

**Figure 7:**
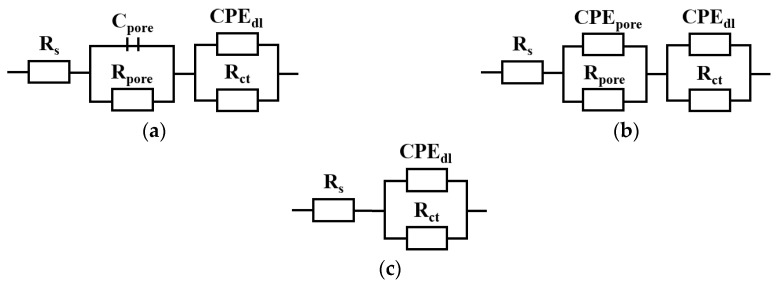
Electric substitute scheme: (**a**) S_is; S_tex; S_PCL/TiO_2_, (**b**) S_PCL; S_tex/PCL/TiO_2_, and (**c**) S_tex/PCL.

The characterization of the interface impedance of the tested samples was performed by approximating the EIS experimental data using physical electrical models of the equivalent circuits. As shown in Figure 7, for the S_tex/PCL samples group, an equivalent circuit with single constants was used to analyze the EIS data, which indicates the occurrence of a single layer. The electrical equivalent circuit model of the textured surfaces with PCL nanofiber layers consists of solution resistance (R_s_), the resistance of the conformal layer (R_ct_), and the capacity of the layer (CPE_dl_). For other samples, an equivalent circuit with two time constants was used to analyze the EIS data, which indicates the occurrence of two sub-layers. In this case, the equivalent circuit consists of C_pore_/CPE_pore_ (capacity of the double-layer porous surface) and R_pore_ (resistance of double-layer porous surface), which are representatives of the electrical porous layer, whereas CPE_dl_ and R_ct_, represent the resistive and non-ideal capacitive behavior of the passive film. In particular, we have the following:

R_pore_—the electrolyte resistance in the porous phase;

C_pore_/CPE_pore_—the capacity of the double-layer porous surface;

R_ct_—the electric charge transfer resistance at the boundary of phases;

CPE_dl_—the capacity used to describe the low-frequency region (11–0.001 Hz).

The mathematical impedance model of the above system is also presented in Equations (2)–(4).

(2) $$Z=R_{s}+\frac{1}{\frac{1}{R_{pore}}+j\omega C_{pore}}+\frac{1}{\frac{1}{R_{ct}}+Y_{\mathrm{d}l}{\left(j\omega \right)}^{n_{dl}}}$$

(3) $$Z=R_{s}+\frac{1}{\frac{1}{R_{pore}}+Y_{1}(j\omega {)}^{n_{1}}}+\frac{1}{\frac{1}{R_{ct}}+Y_{02}(j\omega {)}^{n_{2}}}$$

(4) $$Z=R_{s}+\frac{1}{\frac{1}{R_{ct}}+Y_{01}(j\omega {)}^{n_{2}}}$$

The parameters characterizing the electrochemical response of the surface of the tested samples are provided in Table 4. The samples with the PCL or PCL/TiO_2_ layer exhibited the highest resistance values, affirming that the applied protective layers on the material played a role in enhancing its anticorrosive properties.

### 3.4. Potentiodynamic Test

**Figure 8:**
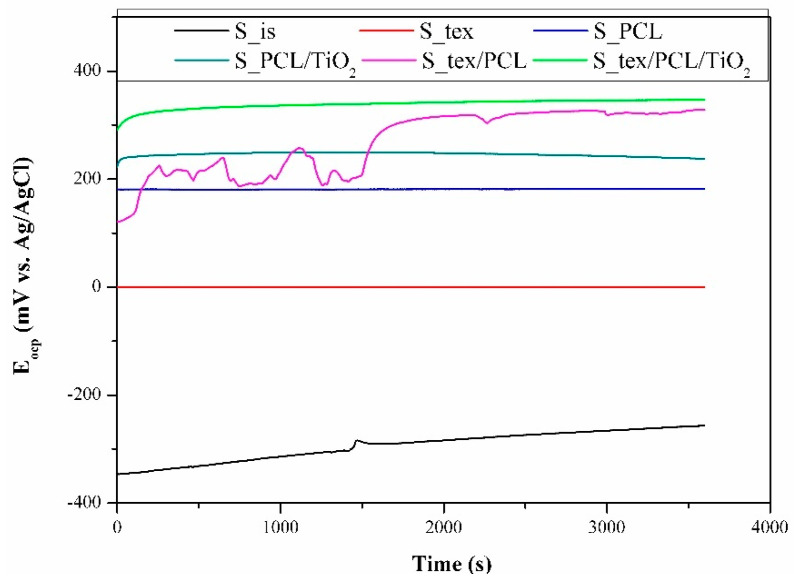
Results of E_ocp_ in time function measurements.

The open circuit potential curves recorded in t = 1 h are depicted in Figure 8. In the case of the samples in the initial state (S_is), the open circuit potential values consistently increased throughout the entire measurement time, reaching more electropositive values without attaining a stable or stabilized state. The positive shift in E_cop_ values indicates an augmentation in the compactness of the passive layer or corrosion products on the samples with time. Samples after the laser-texturing process (S_tex) and samples with a deposited PCL layer (S_PCL) exhibit a similar progression of E_ocp_ curves, showing almost no change over the measuring time. For the S_tex/PCL samples group, the E_ocp_ values begin to increase, and then gradually stabilize. Additionally, up to 1650 s, some oscillation of E_ocp_ was visible, which pointed to some instability of the surface layers, or was due to localized corrosions on the metal surface in the aqueous solution. An initial increase in E_cop_ values with a gradual stabilization was also observed for the S_PCL/TiO_2_ and S_tex/PCL/TiO_2_. However, the steady-state for those samples was reached after approximately 200 s.

The lowest values of E_ocp_ were recorded for the S_is samples group, and the mean value was close to −242 mV vs. Ag/AgCl (corresponding to approximately –45 mV vs. NEH). The recorded values belong to the domains of the passive region of TiO_2_ in the titanium Pourbaix diagram. This means that the alloys form a stable oxide layer of TiO_2_, as we also recorded in our previous article [6,58]. The surface modification of Ti-6Al-4V substrate materials results in shifts in the E_ocp_ value towards more electropositive values, in the following order: S_tex/PCL/TiO_2_ > S_tex/PCL > S_PCL/TiO_2_ > S_PCL > S_tex. For the S_tex samples group, the E_ocp_ value was +10 mV vs. Ag/AgCl (+207 mV vs. NEH), which could fall within the domains of the TiO_3_·2H_2_O region in the titanium Pourbaix diagram. However, the increase in values compared to those obtained for the samples in the initial state can be associated with the head-build effect during the laser-texturing process, promoting oxidation and microstructural changes near the surface. Similar observations were reported by Annamala et al. [59], Yue et al. [60], and Bussoli et al. [61]. In effect, as a result of laser interaction, different titanium oxides could be formed. 

**Figure 9:**
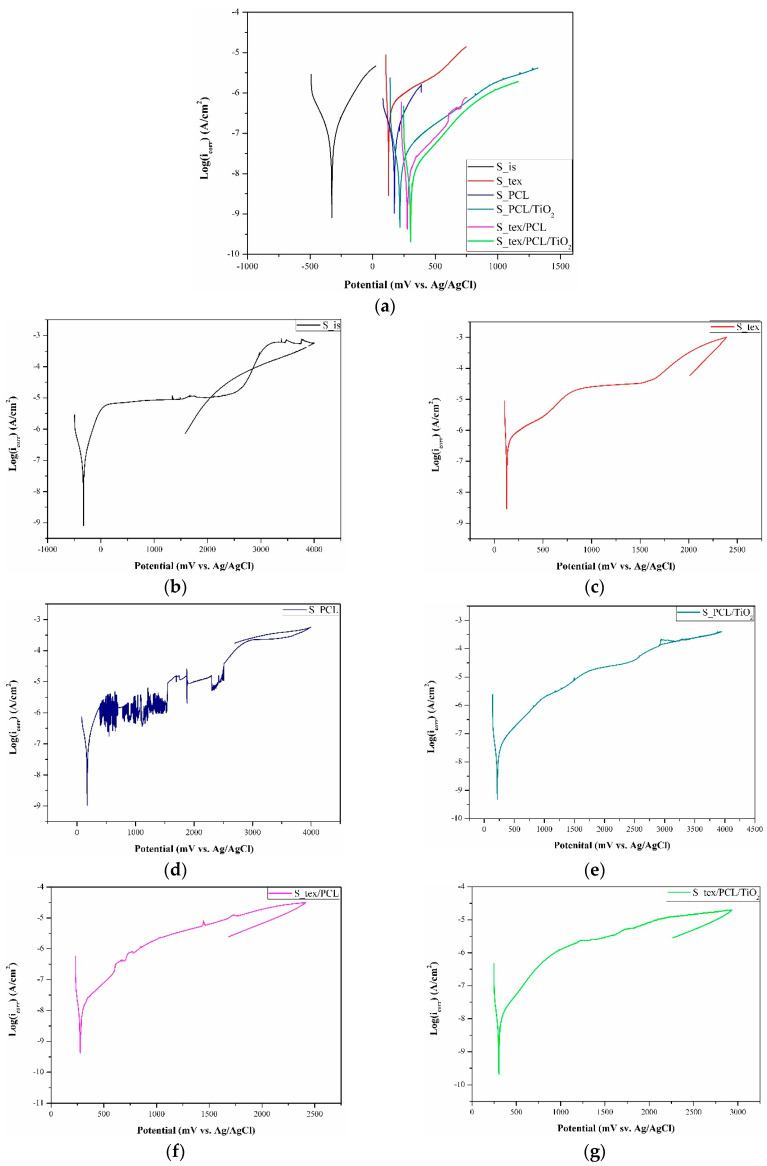
Results of potentiodynamic test: (**a**) Tafles plot for all tested samples, and example potentiodynamic curves for (**b**) S_is, (**c**) S_tex, (**d**) S_PCL, (**e**) S_PCL/TiO_2_, (**f**) S_tex/PCL, and (**g**) S_tex/PCL/TiO_2_.

To evaluate the effect of the surface modification on the corrosion resistance of the titanium alloy, potentiodynamic tests were carried out. The results of the potentiodynamic test for all tested samples are presented in the form of Tafel’s plot and polarization curves in Figure 9. The characteristic corrosion parameters are given in Table 5.

It was found that the registered values of the corrosion potential (E_corr_) confirm the E_ocp_ behavior. The surface modification led to shifts in the values of the corrosion potential to more noble values. Additionally, it was observed that TiO_2_ particles in the nanofiber PCL matrix provide higher values of E_corr_. The most favorable values of E_corr_ were indicated for S_tex/PCL/TiO_2_ and S_tex/PCL, while the lowest values were recorded for the S_tex and S_is samples groups. Analysis of the recorded curves revealed variations in the corrosion resistance based on the surface condition of the tested samples. For the S_is and S_tex samples, plateau regions were recorded from +200 to +2200 mV vs. Ag/AgCl and from +900 to +1600 mV vs. Ag/AgCl, respectively, followed by a steady increase in current densities. Voltammetric curves for both sample groups showed an active-to-passive transition with current densities of about −350 and 300 µA/cm^2^, respectively. Other tested samples displayed a steady increase in the current density without a plateau region. For the S_PCL and S_PCL/TiO_2_ samples, an active-to-passive transition occurred with current densities of about −330 µA/cm^2^, and, for S_tex/PCL and S_tex/PCL/TiO_2_, the transition was with current densities of about −450 µA/cm^2^. In general, the active-to-passive transition refers to the change in the electrochemical behavior of the material. Negative current densities signify the cathodic current, which typically corresponds to reduction reactions and can be associated with the transition from an active to a passive state. Therefore, the more negative the current density is, the more effective the material is in transitioning to a more corrosion-resistant, passive state. The existence of breakdown potential (E_b_) was recorded only for the S_PCL samples group, hysteresis loop. For the other tested samples, the existence of the transpassivation potential was recorded. For samples in the initial state, the transpassivation potential (E_tr_) and corrosion current density (i_corr_) were +1952 mV vs. Ag/AgCl, and 0.033 µA/cm^2^, which correspond to our previous work [6] and literature data. It was found that the laser-texturing treatment worsened the corrosion resistance of the titanium alloy surface. The registered values of E_tr_ were lower than those in the initial state, and the i_corr_ was higher. According to the literature data, conflicting information about the influence of laser texturing on Ti-6Al-4V’s corrosion resistance can be found. For some authors [7,19,62,63], the laser-texturing process leads to an improvement of the corrosion resistance of the Ti-6Al-4V titanium alloy, which can be directly correlated with an increase in the wetting angle, and the grain refinement of laser-textured surfaces. As indicated by Kumari et al. [64], grain refinement improves the passive film formation due to the increased grain boundary density. Contrarily, Grabowski et al. [65] showed a decrease in corrosion resistance after laser texturing Ti-6Al-4V alloy, contributing to increasing roughness. Moreover, Wang et al. [66] investigated the corrosion resistance of Ti-6Al-4V after the laser-texturing process with the micro-groove width in the range of 25 to 65 µm. Depending on texture patterns, different corrosion resistances of the substrate materials were found. Based on the potentiodynamic test only for the samples with a groove width of 35 and 45 µm, an increase in the corrosion resistance was observed. As mentioned above, the laser-texturing process can lead to microstructural changes and increase the passivation ability. However, in some cases, laser texturing can induce crystallography orientation changes, which may influence corrosion susceptibility, especially if the changes result in preferential corrosion along a specific path. In general, titanium exhibits the spontaneous ability to form a robust and uniform TiO_2_ passive layer, which is critical for protecting the alloy against corrosion. A well-formed passivation layer acts as a barrier, hindering the penetration of corrosive agents. However, the laser-texturing process may alter the composition or characteristics of this oxide layer. Changes in the oxide layer can impact the overall corrosion resistance of the material. Additionally, laser texturing may not uniformly passivate the entire surface, leaving some areas more susceptible to corrosion. This corresponds to the E_ocp_ behavior examination, which shows the possible formation of a Ti_2_O_3_ passive layer on the laser-textured surface, which is less stable than a TiO_2_ passive layer. The Ti_2_O_3_ is more prone to further oxidation and can be less protective as a passive layer under certain conditions [67]. The reduced protective capability of the Ti_2_O_3_ passive layer may lead to an increase in implant degradation and the release of metal ions from the implant into the surrounding tissues. Consequently, this may result in chronic inflammatory responses. A decrease in corrosion resistance after laser texturing can be attributed to residual stresses in the material, particularly at the textured regions. These stresses may create microcracks or defects in the surface, providing initiation sites for corrosion and compromising the overall resistance. For other samples after surface modification, an increase in the E_tr_/E_b_ values and a decrease in the i_corr_ values were registered. This indicates an improvement in the corrosion resistance of the Ti-6Al-4V titanium alloy. The most favorable corrosion parameter was registered for the samples with a PCL/TiO_2_ nanofiber layer—S_PCL/TiO_2_ and S_tex/PCL/TiO_2_. First, the better corrosion resistance of those sample groups can be attributed to the barrier effect. The electrospun nanofiber layers can act as a physical barrier, limiting the direct contact between the corrosive environment and the Ti-6Al-4V substrate. The barrier effect was also indicated as a reason for improving corrosion resistance in our previous work [56,57,68,69]. Moreover, according to the literature data [70,71,72], enhanced corrosion resistance has been observed by preventing the diffusion of corrosive ions into the substrate material through the deposition of a PCL-based layer. However, PCL, as a biocompatible polymer, can contribute to the overall biocompatibility of the modified surface. The interaction between the modified surface and biological environments may influence the corrosion behavior and the response of the surrounding tissues. Additionally, the incorporation of TiO_2_ nanofibers in the layers can enhance the formation and stability of a protective oxide layer on the Ti-6Al-4V surface. 

Moreover, an increase in the contact angle and hydrophobic surface properties can provide a better corrosion behavior. Generally, hydrophobic surfaces (higher contact angles) repel water, potentially minimizing the contact between the corrosive medium and the material. However, the influence of the micro- and nanoscale roughness on the wettability and the corrosion resistance must also be taken into account. The surface roughness can impact both the contact angle and corrosion resistance. That is why an analysis of the wettability state presented in Section 3.2 could be helpful in corrosion resistance analysis. Dănăilă et al. [73] showed that the passivation behavior of the Ti-6Al-4V alloy was affected by the surface’s roughness. Researchers have also shown that samples with higher microroughness exhibit lower corrosion resistance. However, sub-microroughness or hierarchical roughness can be beneficial for corrosion resistance. Micro- and nanoscale features may affect the ability of corrosive agents to come into contact with the material, influencing the corrosion process. As was reported in [74,75,76], the air entrapped in surface roughness can prevent aggressive ions from attacking the substrate material surface. In effect, the presence of the hydrophobic surface leads to a shift in the anodic corrosion potential toward more noble values, and both the anodic and cathodic corrosion currents are significantly reduced [77]. Zhang et al. [78] explained that the deposition of a densely packed superhydrophobic layer on a titanium-based substrate material was enough to prevent oxygen from diffusing into the substrate. Moreover, capillarity is an important aspect to consider in order to improve the corrosion resistance of a hydrophobic surface. As indicated by Liu et al. [78], for high contact angle values, water transport against gravity is easy in the porous structure of superhydrophobic surfaces. In effect, as a result of the Laplace pressure, the corrosion solution can be pushed out from the pores. Additionally, in our work, the micro-grooves of the laser-textured samples could be filled with PCL and PCL/TiO_2_ nanofiber layer deposition. Khoshanood et al. [79] indicated the better corrosion behavior of the AZ31 alloy as a result of the deposition hybrid PCL/chitosan scaffold coatings. Catauro et al. [80] investigate the influence of surface modification by the deposition of an inorganic TiO_2_ matrix with different percentages of PCL by the sol–gel method. The electrochemical results pointed to the notion that the coatings have a significant effect in terms of the corrosion potential, as also shown in our work. However, during the drying and curing stages, the sol–gel film may experience cracking and shrinkage. This can compromise the integrity of the coating and reduce its effectiveness, particularly in applications requiring a continuous and defect-free layer. Moreover, coating complex shapes or intricate structures with sol–gel films can be difficult. The electrospinning process eliminates this limitation.

### 3.5. Inductively Coupled Plasma Atomic Emission Spectroscopy

According to the results obtained by ICP-AES it can be seen that laser surface texturing leads to an increase in the amount of titanium and aluminum ions released into the Ringer solution. Ions released from metal implants into a corrosive environment have the potential to trigger inflammatory responses in the surrounding tissues, compromising the overall biocompatibility of the implant. This immune reaction may manifest as localized inflammation and, in some cases, allergic responses, which can range from mild irritation to more severe reactions that may jeopardize the implant’s functionality. Furthermore, the toxicity of certain metal ions could pose risks to tissues, affecting the health of the surrounding anatomical structures. Prolonged exposure to released ions, even at low concentrations, may contribute to chronic health issues and impact the normal healing process at the implant site [81,82,83]. For example, elevated aluminum levels could lead to neurotoxicity and neurological disorders. In addition, aluminum ions can interfere with bone mineralization. Excessive aluminum exposure has also been linked to renal impairment. As is well-known, the kidneys play a crucial role in filtering and excreting ions from the body. The release of aluminum ions may present challenges for renal function [84].

The concentration of titanium ions in the solution after the immersion test (t = 7 days) of the S_tex samples group was close to 60% higher compared to that for the S_is samples group, confirming the lower corrosion resistance of samples after laser texturing as indicated by the EIS and potentiodynamic tests (Table 6). Moreover, the deposition of PCL and PCL/TiO_2_ nanofiber layers on the Ti-6Al-4V titanium alloy surface via the electrospinning method was observed to offer effective protection against the release of Ti-6Al-4V alloy elemental ions into the corrosive environment (human organism). The lowest amount of released ions was observed for the S_PCL/TiO_2_ samples group. Moreover, the protective properties of the PCL or PCL/TiO_2_ nanofiber layers on laser-textured surfaces were observed.

### 3.6. Cytotoxicity Tests

**Figure 10:**
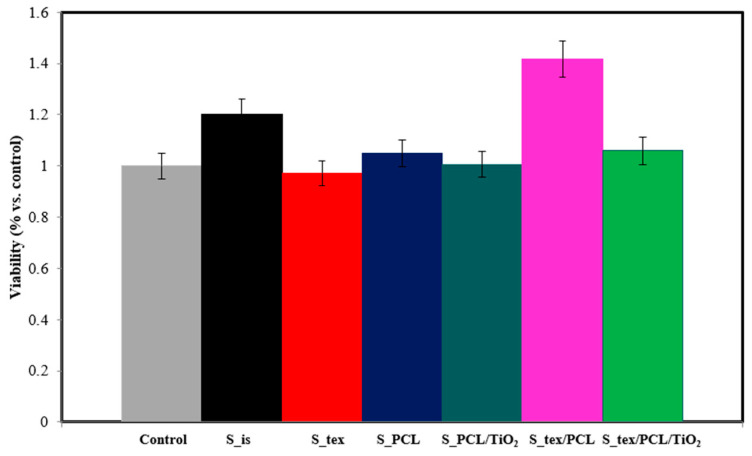
Results of microbiological test t = 72 h.

The results of the microbiological tests for all tested samples are presented in Figure 10. The objective of the test was to evaluate the toxicity of the examined materials intended for potential use as biomaterials. Toxicity is characterized by the ability of a material to disrupt the functioning or cause the death of body cells. Initial signs of these changes manifest in abnormal cell metabolism, and the extent of this phenomenon can be assessed using the MTT test. The results are depicted in graphs illustrating the correlation between the average viability (expressed in %) and the incubation time of cells with the tested material.

After 72 h, for the S_is, S_tex, S_PCL, S_PCL/TiO_2_, S_tex/PCL, and S_tex/PCL/TiO_2_ samples group, the average cell viability of HCT116 cells was 120, 97, 104, 101, 141, and 105%, respectively, compared to the control sample (100%). The obtained results indicate that all samples except S_is and S_tex/PCL do not affect the proliferation of cancer cells. The percentage viability fraction for these materials was approximately 5% of the difference compared to the control sample. This change is too low to suggest that the mentioned materials may have features that stimulate the growth of cancer cells. Any observed changes are likely caused by environmental factors and the accuracy of the test equipment. The only result that differs significantly from the others is the survival fraction of HCT116 cells for the S_tex/PCL sample groups. In this case, an increase in cell proliferation by over 41% was observed compared to the control sample. The duration of the experiment is also important. In the initial period of culture (first 24 h), a decrease in cell viability is often observed. This can be elucidated by the cellular stress induced by the introduction of a foreign element into the medium, such as the tested sample, along with the partial mechanical damage incurred by a specific number of cells during the sample implementation in the culture. After 72 h, the cell count in the culture rises, which can be attributed to the alleviation of cellular stress and the proliferation of cells under favorable conditions in the laboratory. The analysis of the microbiological results after 72 h showed that none of the tested samples exhibited cytotoxicity. It can be stated that the smallest number of cells multiplied after t = 72 h occurred in the case of the S_tex and S_PCL samples group. It can be assumed that the deposition of a layer of PCL and TiO_2_ has a better effect on cell proliferation compared to the deposition of a layer with only PCL.

## 4. Conclusions

Based on the obtained results, it was found that the hybrid surface modification of the Ti-6Al-4V titanium alloy by laser texturing and PCL or PCL/TiO_2_ nanofiber layer deposition using the electrospinning method improves the biofunctional properties of the proposed biomaterial. An increase in the wetting angle and corrosion resistance was observed. Additionally, non-toxic properties of the tested surface conditions were recorded. Future research is planned, to continue exploring these promising findings.

## Figures and Tables

**Table 1:** Parameters of plasma atomic emission spectrometry parameters test.

Parameter		Value (u)
RF power		1.0 kW
Plasma flow		15 (L/min)
Auxiliary flow		1.5 (L/min)
Nebulizer pressure		210 (kPa)
Pump rate		15 (rpm)
Emission lines	Ti	λ = 334.188; 334.941 and 336.122 (nm)
	Al	λ = 237.312 and 396.152 (nm)
	V	λ = 268.796; 292.401; 309.310 and 311.837 (nm)

**Table 2:** List of tested samples with their names and surface conditions.

Name	Surface Condition
S_is	Samples in the initial state—after mechanical grinding and polishing
S_tex	Samples after laser-texturing process
S_PCL	Samples with PCL nanofiber layer de-posted by electrospinning method
S_PCL/TiO_2_	Samples with PCL/TiO_2_ nanofiber layer de-posted by electrospinning method
S_tex/PCL	Samples after laser-texturing process and with PCL nanofiber layer de-posted by electrospinning method
S_tex/PCL/TiO_2_	Samples after laser-texturing process and with PCL/TiO_2_ nanofiber layer de-posted by electrospinning method

**Table 3:** Results of contact angle measurements.

Name	Contact Angle (°)		Surface Free Energy (mJ/m^2^)		
	Distilled Water	Diiodomethane	$\gamma_{S}$	$\gamma_{S}^{d}$	$\gamma_{S}^{p}$
S_is	68.7 ± 2.9	47.0 ± 3.1	36.6	22.7	18.9
S_tex	95.4 ± 3.9	42.0 ± 2.8	39.4	38.1	1.7
S_ PCL	128.2 ± 1.4	17.2 ± 1.1	106.6	85.7	20.9
S_PCL/TiO_2_	137.8 ± 1.2	10.4 ± 0.7	116.4	91.1	25.3
S_tek/PCL	133.3 ± 2.1	13.5 ± 3.4	111.0	88.3	22.6
S_tex/PCL/TiO_2_	137.9 ± 1.5	-	-	-	-

**Table 4:** Results of EIS test—values of resistances.

No	Name	R_pore_ (Ω·cm^2^)	R_ct_ (kΩ·cm^2^)
1	S_is	30	432
2	S_tex	48	393
3	S_PCL	10	885
4	S_PCL/TiO_2_	650	244
5	S_tex/PCL	650	890
6	S_tex/PCL/TiO_2_	-	890

**Table 5:** Results of potentiodynamic test—value of characteristic corrosion parameters.

No	Name	E_corr_ (mV vs. Ag/AgCl)	E_tr_ (mV vs. Ag/AgCl)	E_b_ (mV vs. Ag/AgCl)	i_corr_ (µA/cm^2^)
1	S_is	−301 ± 27	1952 ± 35	-	0.033
2	S_tex	+110 ± 35	1794 ± 41	-	0.052
3	S_PCL	+172 ± 12	-	2290 ± 39	0.019
4	S_PCL/TiO_2_	+220 ± 26	2392 ± 32	-	0.012
5	S_tex/PCL	+275 ± 52	2446 ± 57	-	0.015
6	S_tex/PCL/TiO_2_	+296 ± 44	2652 ± 61	-	0.011

**Table 6:** Results of ICP-AES analysis.

No	Name	Release Ions (mg/L)		
		Ti	V	Al
1	S_is	1.03	0.06	0.07
2	S_tex	1.63	0.06	0.09
3	S_PCL	0.91	0.04	0.05
4	S_PCL/TiO_2_	0.67	0.03	0.05
5	S_tex/PCL	0.80	0.04	0.05
6	S_tex/PCL/TiO_2_	0.82	0.05	0.05

## Data Availability

The data presented in this study are available upon request from the appropriate author. The research results are presented in the article, and the data obtained during the tests are private.
